# An Electrophysiological Model for Assessing Cognitive Load in Tacit Coordination Games

**DOI:** 10.3390/s22020477

**Published:** 2022-01-09

**Authors:** Ilan Laufer, Dor Mizrahi, Inon Zuckerman

**Affiliations:** Department of Industrial Engineering and Management, Ariel University, Ariel 40700, Israel; Dor.mizrahi1@msmail.ariel.ac.il (D.M.); inonzu@ariel.ac.il (I.Z.)

**Keywords:** tacit coordination games, EEG, theta/beta ratio, mathematical modelling

## Abstract

Previously, it was shown that some people are better coordinators than others; however, the relative weight of intuitive (system 1) versus deliberate (system 2) modes of thinking in tacit coordination tasks is still not resolved. To address this question, we have extracted an electrophysiological index, the theta-beta ratio (TBR), from the Electroencephalography (EEG) recorded from participants while they were engaged in a semantic coordination task. Results have shown that individual coordination ability, game difficulty and response time are each positively correlated with cognitive load. These results suggest that better coordinators rely more on complex thought process and on more deliberate thinking while coordinating. The model we have presented may be used for the assessment of the depth of reasoning individuals engage in when facing different tasks requiring different degrees of allocation of resources. The findings as well as future research directions are discussed.

## 1. Introduction

The problem of coordination is that of managing inter-dependencies between the activities of the agents, usually using some form of coordination mechanism or a predefined protocol [1]. Effective coordination often requires establishing and maintaining appropriate common knowledge, beliefs and assumptions that the involved parties share [2,3,4,5]. At its most fundamental level, a coordination problem is one in which two individuals are rewarded for making the same choice from the same set of alternatives. It has been shown that people have an innate ability to successfully play coordination games even when communication is not possible [6,7]; these games are known as “tacit coordination games”, and while humans are highly capable of succeeding in them, computers are still incapable of performing this task. This gap results from the fact that game theory, which studies the interaction between rational decision makers, cannot successfully predict the choice that human players will make when faced with several alternative solutions in tacit coordination games. This is because tacit coordination games involve multiple Nash equilibria, and currently, game theory cannot evaluate the likelihood of preferring one Nash equilibrium over another, resulting in its inability to accurately explain the choice of humans. In these games, particular Nash equilibria seem to constitute ‘focal points’ (or ‘Schelling’s points’) on which the players’ expectations converge (e.g., [6,7,8,9]). 

Although game theory cannot predict the choice of a focal point solution, Schelling postulated that focal point selection relies both on imagination and logic. Thus, Schelling suggests that imagination allows people to instinctively rely on certain cues to recognize some focal points; however, this is not sufficient, and they also rely on logic. Thus, there are two types of reasoning involved: one is more instinctive in nature while the other involves reasoning. Bacharach developed the reasoning concept by introducing variable frame theory [10,11,12]. This theory posits that players use noticeable frames to reframe the game. Then, by applying a reasoning process based on payoff dominance and symmetry disqualification rules, they try to reach a variable frame solution [11]. Thus, while Schelling emphasizes the reliance on instinctive cues, Bacharach’s theory entails a more in-depth reasoning process.

This distinction between a more intuitive process and a more deliberate one has been studied using the dual process cognitive framework (e.g., [13,14,15]). Achieving coordination involves both types of processes, intuitive and deliberate, which interact with each other and are implicated in effective coordination, which relies on common knowledge [16,17,18]. Thus, the convergence on the same solution might be highly automatic when heuristics are being used or, on the other hand, mostly deliberate when players employ [19] deeper reasoning processes to infer what might be the line of reasoning of the co-player. This distinction between a deliberate (system 2) and automatic process (system 1) is analogous to the distinction between thought–analytical processes and fast heuristics [6,20] and is in line with Tversky and Kahneman’s suggestion of human-bounded rationality [21,22]. In previous studies, the authors of this paper have shown that there are individual differences in the coordination ability of different people [23,24,25,26]; that is, some people are better coordinators than others. With that in mind, in this study, we will examine whether there is a correlation between coordination ability and the depth of reasoning used by players. 

To that end, in this study, we have used a pure tacit coordination game where communication between players is not allowed, and therefore, this experimental environment is appropriate for examining the depth of reasoning in the context of dual process theory [27] during cooperative decision making [7,25,28,29,30]. To measure depth reasoning, we recorded Electroencephalography (EEG) from participants while they were performing a tacit coordination game. From the EEG, we have extracted an electrophysiological index, the Theta-Beta Ratio (TBR), which are frequency bands that provide an index of oscillatory activity associated with cognitive load [31,32,33]. The TBR enabled us to examine whether there is a correlation between coordination ability and cognitive load. We have shown that better coordination ability is associated with higher cognitive load. This finding indicates that the deliberate process (system 2) is probably more dominant in tacit coordination compared to automatic processes (system 1). This electrophysiological index may therefore be integrated into decision support systems, which aim to estimate the degree of reliance on each of the systems (system 1 and system 2) during cooperative decision-making scenarios [34]. In this study, we have used statistical methods, which enabled us to estimate the relationship between behavioral coordination ability and an electrophysiological measure as an index of the magnitude of reliance on each of the two systems. The regression model enables the estimation of the magnitude of the electrophysiological response reflecting the depth of reasoning (intuitive or more deliberate).

## 2. Materials and Methods

### 2.1. Participants

The participants were 7 males and 3 females, undergraduate students at Ariel University (right-handed, mean age = 26 (years), SD = 4). Following the verbal explanations, participants read a written instructions file displayed on the computer screen.

### 2.2. EEG Recording and Preprocessing

A 16-channel g.USBAMP (g.tec, Schiedlberg, Austria) was placed on the scalp following the 10–20 system for EEG recording (using OpenVibe [35]) at a sampling frequency of 512 Hz. Impedance of all electrodes was kept below 5 K [ohm]. Data processing was performed using the EEGLAB package [36]. Preprocessing stages consisted of band-pass filtering [1,32] Hz, followed by a notch filter of 50 Hz. Independent component analysis (ICA) was applied on the filtered signal (e.g., [36,37]). Finally, the data was re-referenced to the average reference and down sampled to 64 Hz following baseline correction. Epoch windows of 1 s were extracted in each of the trials relative to game onset. Response time was measured using the OpenVibe recording software with a 5 (ms) resolution from game onset.

### 2.3. Experimental Design

The methods used to extract the TBR [32,38,39,40,41] from the EEG are based on exactly the same preprocessing pipeline and Discrete Wavelet Transform (DWT) [42,43] calculations detailed in a previous study [26]. EEG was recorded by using g.USBAMP from 16 channels at a sampling frequency of 512 [Hz]. Ten Ariel University students (right-handed, mean age = 26 (years), SD = 4) were recruited for this study. This study comprised three experimental conditions. In the first condition, EEG was recorded at rest while subjects were requested to focus on a cross overlayed over a grey background that was displayed on the screen. In the other two conditions, participants played a game in which they were requested to select a word out of a string of four words. In the second condition, the picking condition, participants were asked to freely pick a word representing an object out of the string (e.g., {“Tennis”, “Volleyball”, “Football”, “Chess”}) while in the third condition, the coordination condition, the participants were requested to coordinate their choice of a word with an unknown partner without any communication. Picking and coordination each comprised 12 games each, including strings of four words written in Hebrew (see Appendix A). The game order was randomized in each condition, but the word order within each string remained constant. The experimental conditions were presented at the following fixed order: resting state, picking and coordination. This presentation sequence was predetermined so that we could extract a measure of baseline TBR not affected by a preceding task-related cognitive activity (see [44]) and to prevent the contamination of the picking condition with the coordination rules. Between each condition, there was a three-minute break. In the picking condition, the participant had to randomly pick one word out of the string, whereas in the coordination condition, the participants had to try and choose the same word as the co-player. Successful coordination might have occurred when both players converged on the same word [6,28].

### 2.4. Measures

We measured coordination ability both at the individual and group level of analysis. The individual coordination ability (iCA, [23,24,25,26]) was computed based on the TBR and was computed for each of the participants during the engagement in the series of 12 games. The TBR is known to covary with activity in the executive control and default mode networks [38,39]. TBR decreases as cognitive load increases and vice-versa [38,39]. To estimate cognitive load in this study, we used power-based features extracted by employing discrete wavelet transform (DWT) [42,43,45]. For each of the EEG epochs, we estimated the relative average power and then calculated the ratio between the relevant bands [46,47]. Collectively, previous studies indicate that TBR is associated with various psychological functions that are likely to be meditated by subcortical mechanisms, including self-regulation and motivational control [39]. At the group level, we computed the coordination index (CI, [7,23,28]), which reflects the difficulty level of each of the 12 games and is an aggregated measure across the participants. Briefly, the iCA reflects the total number of games in which each player was able to coordinate their responses against the entire population. The higher the player’s iCA value (ranged in [0, 1]), the higher their coordination ability. The CI index reflects the probability that two distinct individuals, chosen at random without replacement from the set of N individuals, choose the same label. This measure ranges between 0 (all of the participants chose a different label) to 1 (all participants selected the same label). The formal definition of each these measures is given in our previous studies (iCA, CI [7,23,24,25,26,28]).

## 3. Results

In this section, we first compared the TBR among the three conditions (resting-state, picking, coordination), then we examined whether there is a correlation between iCA (individual coordination ability) and TBR, as well as between TBR and CI (coordination index, the difficulty level of each game). 

### 3.1. Differences in TBR among the Three Conditions

To examine differences in the distribution of TBR among the three conditions, we conducted a one-way repeated-measures ANOVA with Condition (resting-state, picking, coordination) as the dependent variable. To differentiate between the three experimental conditions, statistical analyses included the six frontal and prefrontal electrodes (Fp1, F7, Fp2, F8, F3, and F4), since previous findings have shown that anterior and frontal midline areas are sensitive to workload manipulations (e.g., [31,48,49,50,51]). 

The results of the ANOVA model showed that there was a significant effect of the experiment state on the TBR (F(2,837) = 5.66, *p* < 0.01). Table 1 presents the quantiles of the TBR distribution for each condition.

The cognitive load of the player is inversely related to the TBR value; that is, the lower the TBR value, the greater the cognitive load and vice versa. A gradual decrease in the median TBR values can be observed from resting-state to the coordination condition, indicating that cognitive load is the highest in the coordination condition and the lowest in the resting-state condition. This result corroborates the experimental manipulation since it suggests that the different conditions induce different levels of mental effort. To statistically evaluate the significance of the pairs of relationships, we continued the calculation by using the Tukey post-hoc test [52]. The difference in TBR was found to be statistically significant in all the pair-wise comparisons (resting state-picking and picking-coordination *p* < 0.05; (resting state-coordination *p* < 0.001)). Notably, the size effect of the comparison between resting-state and the coordination condition was larger than the comparison between the resting-state and the picking condition. This finding might indicate that system 2 was implicated to a greater extent in the coordination condition than in the picking condition, which probably relied on fast heuristics and less deliberate thinking (system 1).

### 3.2. The Relationship between CI and TBR

In previous studies [23,24,25], we have shown that there are individual differences in tacit coordination ability (see [23,24,26]). Moreover, we have shown that the higher the iCA, the smaller the TBR, which is associated with a higher cognitive load. Therefore, there is a linear negative relationship between TBR and iCA [26]. In this section, we analyzed the difficulty level of the 12 games at the group level of analysis. The 12 games are different in terms of the difficulty to identify the focal point. For example, in some games, there might be a single prominent focal point solution, whereas as other games might include multiple focal point solutions similar in their degree of saliency. Therefore, a player who might be a good coordinator will not invest a large amount of cognitive resources when the game is relatively easy. Thus, to examine the effect of game difficulty, we quantified the difficulty of each of the 12 games by calculating the CI index (e.g., [7,28]). For each game board, we averaged the individual across participants. In a previous study [26], the authors of the current paper showed that all frontal and pre-frontal electrodes demonstrated similar trends within a regression model describing the relationship between coordination ability and TBR. Moreover, the F4 channel has shown the strongest statistical effect in the regression model. Consequently, further data analysis was based on this electrode.

We first calculated the CI index based on the responses of all participants in the experiment, as presented in Table 2. Game #9, the most difficult game to coordinate with a CI of 0.178, contained the names of four European capitals. Apparently, according to the spread of responses, there appeared to be no prominent focal point, and therefore, in this, game coordination was rather difficult.

To model the relationship between the CI index and the TBR, we calculated a first-order linear regression model on the F4 channel. The linear regression equation together with the relevant measures of model fit can be seen in Equations (1) and (2).

**Figure 1:**
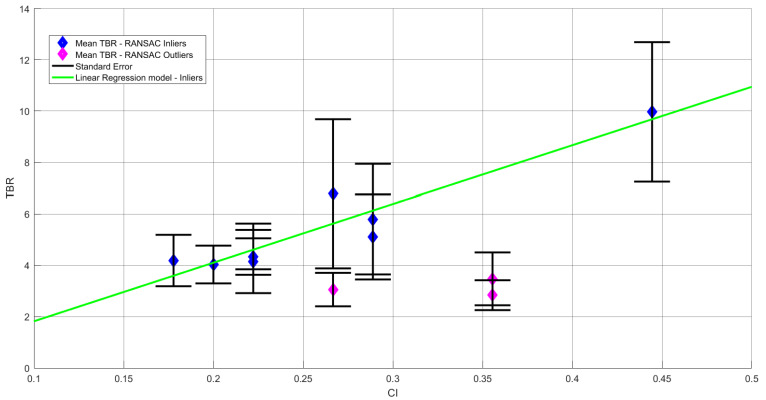
CI effect on TBR—regression model—channel F4.

We identified the outliers in the data using the Random Sample Consensus (RANSAC) algorithm (e.g., [53,54,55,56]). The main advantage of the RANSAC algorithm is its robustness to noise. The output of the RANSAC model indicated that the inliers’ data points accounted for 75% of the original data (9/12 samples). Next, the linear regression was run only on the data set including inliers. The linear regression equation and the measures of model fit can be seen in Equations (1) and (2), respectively, where *X* denotes the CI measure and *Y* denotes the TBR. The positive relationship between CI and TBR is displayed in Figure 1.

(1) Y=−0.5093+22.9318·X

(2) $$R^{2}{}_{inliers\;}=0.8879,\;F_{statistic\;}=47.546,\;p<0.001,\;{VAR}_{error\;}=0.54$$

This positive relationship between CI (a measure of game difficulty) and the average TBR (which has an inverse relationship with cognitive load) indicates that easier games are associated with a lower mental effort.

### 3.3. The Relationship between Response Time and iCA

**Figure 2:**
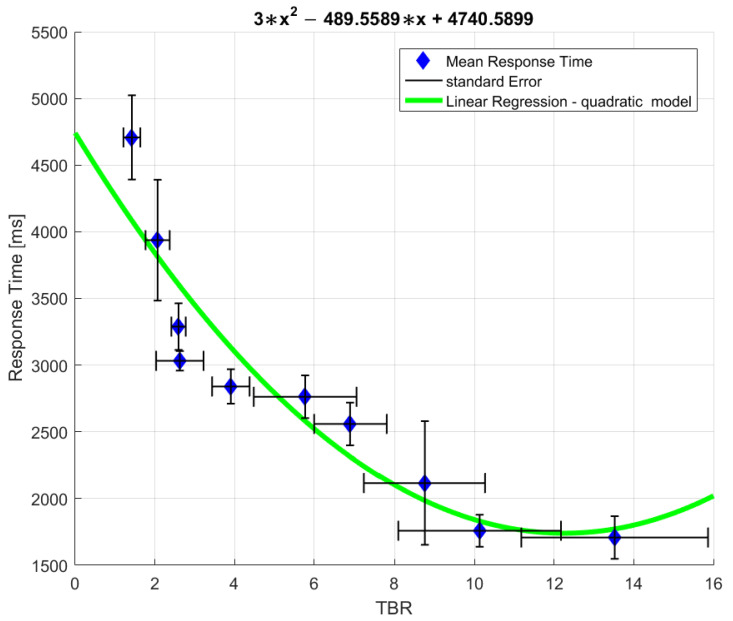
Quadratic regression model.

In this section, we wanted to see whether there was also a relationship between response time (RT) and TBR that was calculated for the F4 electrode. A negative relationship corroborates that a more deliberate thinking is applied in coordination compared to picking and therefore that system 2 is probably more involved in that condition. Figure 2 displays the negative relationship between TBR and iCA. A quadratic regression model demonstrated the best fit to the data (*p* < 0.001). This relationship entails that the lower the TBR, that is the higher the cognitive load, the longer the RT. The longer RT associated with the coordination condition strengthens its association with system 2 since it involves a more complex thought process in contrast to the reliance on fast heuristics (system 1) [57].

## 4. Discussion

The goal of this study was to examine the TBR, a measure of cognitive load, in the presence or absence of tacit coordination. We have first shown that the TBR can distinguish between resting state and the other task conditions (picking and coordination). The largest difference was found between the resting-state condition and coordination, which requires higher amounts of mental effort relative to picking. By employing a regression model, we have demonstrated that there is a relationship between individual coordination ability and the TBR magnitude. Moreover, we have shown that the TBR is sensitive to the difficulty level of the game. Overall, the findings of this study show that TBR is negatively correlated with individual coordination ability and that the level of game difficulty is positively correlated with the average TBR [26,32,40,41,58].

The changes in the TBR are related to both variations in beta and theta bands. Previous findings show that the beta band is considered to be related to top-down processes and is associated with increased mental effort during the performance of mental tasks. On the other hand, theta activity is considered to be implicated in bottom-up automatic processes and lower mental vigilance. Together, previous findings support the suggestion that low TBR is associated with top-down processes and with increased connectivity in the executive control network (ECN), whereas high TBR is related to uncontrolled thought and increased connectivity in the default mode network (DMN). Electroencephalography theta/beta ratio covaries with mind wandering and functional connectivity in the executive control network [32].

To the best of our knowledge, this is the first study showing a correlation between individual coordination ability and TBR. Our findings might suggest that tacit coordination in comparison to picking relies more on system 2 than on system 1. This finding is compatible with the level-k theory, which defines picking as level k = 0 and coordination as level k > 0 [59,60,61,62]. In previous studies, we have shown that considerable variability exits in behavioral coordination ability exists between players [23,24,25,30,63,64,65,66]. In the current study, we have shown that as coordination ability increases the TBR which reflects cognitive load, decreases.

Our findings may seem to contradict previous findings related to both behavioral and neurophysiological findings. Specifically, some previous studies have shown that more efficient performers of a complex task use cognitive resources more efficiently compared to weak performers [67,68,69,70,71,72]. In contrast, in the current study, it seems that good performers relied more on deliberate thinking and invested more cognitive resources in relation to weak coordinators. However, this may be because TBR measures the relative change between the energies of two EEG frequency bands and not is not a measure of absolute energy. In addition, it could be that TBR is a measure of executive control [73] and does not reflect the efficiency of the brain circuits.

There are several avenues for future research. First, it would be interesting to examine TBR magnitude modulations in other contexts not related to tacit coordination, such as resource allocation task (e.g., [30,74,75]) and optimal stopping problems (e.g., [76,77]). Second, our previous studies have shown that the cultural background of players as well as their individual preferences may greatly affect the strategies players choose in coordination games [25,65,78]. For example, it was previously shown that highly prosocial participants and highly individualistic ones are fast responders (either they cooperate or defect, respectively) [79]. Hence, the effect of factors associated with both cultural differences and personal characteristics on the TBR and other electrophysiological indices should be further examined. Finally, the findings of this study should be tested in the context of other tacit coordination games in other domains, such as games with spatial cues and object selection [9,29,63,80].

Overall, the findings of this study might be important in screening scenarios, for example, in the context of employee assessment. Some tasks require more deliberate thinking, while others require more prompt decision making, where the decision maker needs to decide in real time. Therefore, electrophysiological indices such as the TBR may provide an essential insight regarding the cognitive style of potential candidates for different tasks requiring varying degrees of reliance on cognitive resources [81,82].

## 5. Conclusions

In the current study, we have shown that as coordination ability increases, the TBR, which reflects cognitive load, decreases. Hence, one possibility is that good coordinators do not rely on heuristics and intuitive thinking but rather on more deliberate thinking processes [83], with which magnitude is correlated with the difficulty level of each game (see Figure 1). These findings corroborate previous research showing a relationship between TBR and executive control [31] and that coordination relies more on deliberate thinking [57]. On the other hand, it is possible that trying to infer the intentions of the co-player, which necessitates the reliance on additional resources, may eventually lead to the reliance on heuristics since the enhanced mental effort might interfere with a more analytical decision process [84]. Therefore, it could be that those players associated with high iCA values, i.e., those with a higher cognitive load during task performance, revert to using system 1 at some point before reaching a decision regarding their preferred choice of a word. These possibilities should be examined in future studies.

## Figures and Tables

**Table 1:** Theta-Beta Ratio (TBR) distribution by the experimental state—main percentile values.

	**Resting State**	**Picking**	**Coordination**
25th percentile	2.5332	2.2242	1.5009
**Median**	**4.6337**	**4.1214**	**2.9274**
75th percentile	8.3537	7.7705	5.4127

**Table 2:** Coordination Index (CI) values of the game boards in the experiment.

Game Number	1	2	3	4	5	6	7	8	9	10	11	12
CI	0.288	0.222	0.222	0.200	0.355	0.266	0.444	0.288	0.178	0.266	0.222	0.355

## Data Availability

The data presented in this study are available on request from the corresponding author. The data are not publicly available due to the privacy of experiment participants.

## Appendix A. Tacit Coordination Game List

The full game list is presented in Table A1. It should be noted that all participants were native speakers of Hebrew, and therefore, the words in each of the games appeared in Hebrew.

**Table A1:** Experimental game list.

Game Number	Option 1	Option 2	Option 3	Option 4
1	Water	Beer	Wine	Whisky
2	Tennis	Volleyball	Football	Chess
3	Blue	Gray	Green	Red
4	Iron	Steel	Plastic	Bronze
5	Ford	Ferrari	Jaguar	Porsche
6	1	8	5	16
7	Haifa	Tel-Aviv	Jerusalem	Netanya
8	Spinach	Carrot	Lettuce	Pear
9	London	Paris	Rome	Madrid
10	Hazel	Cashew	Almond	Peanut
11	Strawberry	Melon	Banana	Mango
12	Noodles	Pizza	Hamburger	Sushi
